# Can digital transformation promote the green innovation quality of enterprises? Empirical evidence from China

**DOI:** 10.1371/journal.pone.0296058

**Published:** 2024-03-11

**Authors:** Yang Huang, Chau-wa Lau

**Affiliations:** 1 School of Business, Macau University of Science and Technology, Macau, China; 2 School of Management, Jinan University, Guangzhou, China; Shanghai Business School, CHINA

## Abstract

Digital transformation constitutes a crucial component of the digital economy and represents a microcosmic manifestation, playing a vital role in advancing enterprise sustainable development from the perspective of green innovation quality. Using the panel data of Chinese listed companies from 2011 to 2020, the study examines the impact of digital transformation on the quality of green innovation. The study finds that digital transformation significantly increases the green innovation quality of enterprises. Moreover, the positive effect of digital transformation on green innovation quality is strengthened by the executive with digital knowledge experience and in regions with high-level intellectual property protection. The study findings contribute to digitalization research and the literature on green innovation, and provide suggestions for managers and policymakers seeking to improve the quality of environmental sustainability through digital transformation in developing economies.

## 1. Introduction

China, as a developing nation, grapples with diverse manifestations of industrial pollution [1]. The escalation of pollution-related challenges has prompted heightened cognizance amongst governments and commercial entities regarding the significance of striking a balance between environmental sustainability and economic growth [2]. Green innovation is perceived as a potent instrument for tackling environmental pollution and fostering economic expansion [3, 4]. However, despite its recognized advantages for sustainable development, many enterprises are reluctant to adopt green innovation [5]. Green innovation strategies tend to be costly and risky, resulting in lower returns for enterprises from green innovation than other projects [6]. Moreover, the Chinese government’s innovation policies and catch-up strategies have created many "patent bubbles" where the number explosion does not represent actual improvements in innovation [7]. If a “green patent bubble” exists in China, the proliferation of green patents with declining quality will be detrimental to the green economic transformation. Therefore, how to promote high-quality green innovation to improve the sustainability of enterprises is an essential issue.

Prior environmental sustainability research has explored why some enterprises engage in green innovation, focusing on the role of stakeholder pressure [8], regulatory forces [9], and industrial factors [10]. However, the prior literature fails to examine the impact of digital transformation on the quality of green innovation. Although some efforts have been made in the existing literature to investigate the association between digital transformation and enterprises’ green innovation [11], the prior research has no consensus on the above theme. While some researchers have identified a positive relationship between digital transformation and green innovation [12], others have not observed a significant interrelation between the above relationship [13]. Given the constraints of limited resources and a delicate environment, the long-term progression of China’s economy hinges on its green innovation capabilities. Moreover, the significance of diverse green innovation patents varies, indicating that assessing the impact of digital transformation on green innovation based on the number of patents may be biased [14]. For example, studies have shown that the purpose of innovation is not limited to improving an enterprise’s competitiveness. Enterprises may use strategic innovation to restrict competitors from filing similar patents, and signal stakeholders’ influence [15].

This study speculates that scholars have different views on this issue for several reasons. First, some studies do not distinguish between green quantitative and qualitative innovations [16]. This study argues that it is important to distinguish them because the types of green innovation play different roles between digital transformation and sustainability performance [17], whether digital transformation enhances green innovation may be examined from the perspective of green innovation quality. Second, considering green innovation’s complexity and long-term nature, the adopted research model neglects some critical conditions, including the moderating factors of internal executives’ digital knowledge experience and the role of external intellectual property protection. Therefore, integrating digital transformation and green innovation quality into a comprehensive research framework is based on the perspective of internal and external knowledge.

This study makes the following contributions to the existing literature. First, this study contributes to the literature on green innovation by investigating the impact of digital transformation on green innovation quality. Although green innovation quality is critical to addressing environmental issues, our understanding of its determinants has focused on firm-level characteristics or the regulatory environment. This study focuses on the influence of digital transformation from an organizational perspective, which is an essential factor influencing the quality of enterprise innovation [18]. Second, this study provides boundary conditions for the relationship between digital transformation and green innovation quality by considering the moderating effect of internal and external knowledge. Based on the upper echelon’s theory and institutional theory, this study moderates the impact of the above relationship by introducing two variables (eg., executive digital knowledge experience and intellectual property protection), representing enhanced managerial discretion and green innovation motivation of executives. On the one hand, upper echelons theory focuses on the level of executives and emphasizes the empirical support of executives’ digital knowledge to the digital transformation process, which enhances enterprises’ motivation for green innovation quality. On the other hand, external governance, such as intellectual property protection, is the institutional force regulating enterprises’ rational use of resource allocation [19]. Therefore, this study reveals a more comprehensive picture of the relationship of this research.

## 2. Literature review

### 2.1. Micro-impact of digital transformation on the enterprise

Emerging digital technologies facilitate enterprise digitization strategies and profoundly reshape business functions [20]. Digital transformation represents an organizational shift underpinned by digital technologies, digital products, and digital platforms. This involves integrating digital technologies into business operations with the aim of value creation and productivity enhancement. The application of digital technologies is predicted to trigger green development within organizations [21]. Digital transformation emphasizes the extensive usage and communication technologies within environmental systems, resulting in structural alterations across various domains in enterprise [22].

Research has concentrated on the positive effects of digitalization on enterprise operations. Prior scholars have studied the micro-level impacts of digital transformation on enterprises from various perspectives. On the one hand, digital transformation can alleviate enterprise financing constraints and improve the corporate financing environment. In recent years, technologies such as artificial intelligence, and blockchain have emerged. These technologies can help financial institutions tap users, evaluate information, improve efficiency, and optimize financial services, further promoting the leapfrog development of fintech [23] and providing a new solution to the financing constraint problem [24]. As Begenau et al. (2018) [25] point out enterprises with a high degree of digital transformation can obtain lower financing costs. Thus, digital transformation reduces financing constraints.

On the other hand, digital transformation can improve the market environment for enterprises’ production and operation. Digitization can increase market efficiency and reduce transaction costs. In this case, improvements in digital infrastructure can increase social welfare by improving market pricing efficiency between different regions [26]. Internet technologies expand the geography and space in which goods are exchanged, reducing transaction costs and increasing transaction efficiency [27]. However, other studies have shown that enterprises face a more complex innovation environment. The broad complementarity of different technological innovations poses new challenges for innovators’ coordination and market design [28]. In response, developing appropriate policies around market competition, regulation, intellectual property protection, and consumer privacy is essential to drive the digital transformation of the economy [29]. In addition, the reputation generated by sustainable corporate development may offset these digital disadvantages [30].

### 2.2. Digital transformation and green innovation

Prior studies reveal a lack of the consensus regarding relationship between digital transformation and enterprise green innovation. On the one hand, enterprises’ application of digital technologies can accentuate their competitive advantage in green innovation [31], although a comprehensive analysis of the underlying impact mechanisms remains elusive. The digital transformation triggered by industrial information technology updates can improve the efficiency of information sharing and facilitate knowledge accumulation, thus enhancing enterprise green innovation [32]. Fernando and Wah (2017) [33] suggest that digital technologies positively impact green innovation and that innovation benefits can cover enterprises’ input costs. On the other hand, digital technology advances can drive enterprises to reacquire production equipment, but during the transition phase of a digital transformation, it will increase resource extraction and energy loss to be able to increase production, which may reduce the firm’s green innovation activities [34]. However, it can lead to decreased centripetal force and insufficient inter-industry linkages, thus making it challenging to promote inter-industry green innovation chain breakthroughs [35].

Regarding the influencing factors of corporate green innovation, Hojnik and Ruzzier (2017) [36] argue that they include command-and-control policies, market-based policies, and corporate organizational structures. Within this context, mandatory regulatory policy measures encompass voluntary emissions abatement initiatives, energy governance, governmental financial support, and environmental compliance enforcement [37]. Conversely, market-oriented regulatory approaches involve carbon emissions trading, environmental rights transactions, and related mechanisms [38]. The organizational structure involves corporate governance mechanisms, environmental quality management systems, and stakeholder pressure [39].

### 2.3. Literature concludes

Research on the economic consequences of digital transformation and green innovation has not yet reached a consensus in academia. On the one hand, few prior studies have focused on the heterogeneity between the quantity and quality of green innovation [40]. Most studies use the number of patents to characterize the quantity of innovation and rarely discuss the quality of green innovation. On the other hand, as the mainstream literature shows, digital transformation has become a powerful driver of corporate green innovation performance [41]. Digital transformation is an effective response for enterprises in the era of low-carbon development. However, there is little research on the theoretical mechanisms of digital transformation on enterprise innovation, empirical research that provides an in-depth analysis of the quality of green innovation in enterprises. How to stimulate the vitality of green innovation and achieve a "win-win" situation between enterprises’ green competitiveness and the ecological environment in the Chinese context?

## 3. Research hypothesis

### 3.1. Digital transformation and green innovation quality

In the digital transformation process, enterprises can use the leading edge of digital technology to achieve strategic goals of resource conservation and environmental protection and to help them achieve a higher level of green innovation. This study argues that the reasons for digital transformation to enhance the quality of green innovation are specified below for illustration.

First, digital transformation helps enterprises understand consumer needs, improving the efficiency of green processes and the differentiation advantages of green products, thereby contributing to the improvement of green innovation quality. Consumer resistance to green new products can suppress the spread of green products. For example, when consumers have a high level of resistance to a certain product innovation (such as green innovation), the peak sales of that product will be lower [42]. On the contrary, an increase in consumer demand for green products will promote the processing and diffusion of green products [43]. In addition, according to Porter’s five forces model [44], when consumers increase their demand for a particular green product, it will increase the differentiated competitive advantage of the product in the market. Green innovation is challenging to generate significant economic benefits in the short term, but it can help companies provide solid technological security and earn a good reputation, thus enhancing their long-term competitive advantage [45]. The essential digital transformation features are reflected in green development, which can provide intrinsic motivation to enhance the quality of green innovation. As consumer preferences evolve towards environmental protection, enterprises’ focus on environmental protection and green products provides a basis for enterprises to leapfrog in green innovation [46], thereby addressing stakeholders’ environmental protection requirements. Digital transformation aids firms in more accurately grasping the green needs of consumers. It plays a vital role in enabling businesses to adjust product schemes timely in response to consumer demands, seize opportunities, elevate services, and enhance green innovation technologies. This, in turn, provides guidance for high-quality green innovation within the firm. Therefore, the development of digital technology stimulates consumers to pursue diverse product needs and creates a two-way communication pattern between product supply and demand [47]. The development of digital technology facilitates consumers to pursue diverse product needs. For example, by implementing environmentally conscious design principles in manufacturing systems, enterprises can reduce environmental and production costs by incentivizing optimal decision-making [48].

Second, based on agency theory, digital transformation enhances information transparency and reduces the principal-agent problem [49]. On the one hand, introducing digital technology talents such as the Internet is essential for firms to enjoy the knowledge dividend. According to innovation diffusion theory, knowledge spillover is influenced by geographic space and decays with distance. However, with the rise of digital technologies such as mobile Internet and cloud computing, digital talents help enterprises break spatial constraints and realize real-time information transmission. The efficiency of information exchange is enhanced [50]. In contrast, the rapid collection and aggregation of industry and market information can provide conditions for innovative practices [51]. Digital transformation satisfies a firm’s need for information acquisition, and effective allocation of information elements furnishes momentum for a green innovation quality. Data, an informational resource, strengthens knowledge spillover via information acquisition and interaction, thereby enhancing the organization’s capacity for knowledge learning [52]. With the advantages of information dissemination, data creation, and information sharing, digital transformation facilitates the resolution of contradictions between supply and demand. It enables the construction of cloud-based information exchange platforms to ensure the smooth flow of information [53], offering information sources for high-quality green innovation.

Furthermore, with the matching combination of talent and digital technology, resource allocation efficiency will be improved, and more management innovation and application innovation will be carried out while introducing digital technology changes [54]. The development of digital infrastructure will prompt companies to rely on digital technology to use Internet thinking and improve the internal management model of the organization. In addition, digital transformation is considered to combine new-generation information to trigger significant change. Digital transformation drives the green technology orientation of companies and improves the quality of green innovation. According to the sustainable development goals, high-quality green technologies can improve the economy’s response to climate change and the global environment [55], reducing pollution emissions and implementing green innovation quality to drive corporate sustainability, including redesigning complex processes within the company and developing green innovation capabilities, thus improving the quality of green innovation.

In sum, enterprises’ use of digital technology to help shareholders track critical metrics and up-to-date financial data makes the management process more transparent. It helps reduce opportunistic speculative behavior by management, thus effectively mitigating agency conflicts between management and shareholders. Companies are willing to invest their limited resources in high-quality green innovation, and obtain considerable economic returns. Therefore, the following hypothesis is proposed.

**H1: Digital transformation has a positive impact on corporate green innovation quality**.

### 3.2. The moderating role of executives’ digital knowledge experience

Upper echelons theory suggests that executives’ characteristics, such as cognitive structure and values, map to corporate decision-making and strategy choices [56]. Specifically, executives’ educational experiences and functional backgrounds influence the formation of their cognitive structures and values, which impact the firm’s strategic decisions. This study suggests that executives with digital knowledge experience are more likely to support using digital technology [57]. This research argues that executives with digital knowledge experience domains exhibit agility in seizing developmental prospects and identifying cutting-edge tendencies [58]. Such individuals can convert their accumulated proficiency and experience into decision-support mechanisms, thereby augmenting organizational adaptability and ingenuity in intricate settings, ultimately ameliorating green innovation quality.

First, the ultimate goal of digital transformation is to achieve a global transformation of business models and value creation processes, which requires a long-term commitment from the enterprise [59]. Executives with digital knowledge experience have both a technology background and management function, and have a deep understanding and knowledge of technology application and business operation, which enable them to expect the time required for digital transformation strategy reasonably and make long-term strategic planning [60].

Second, data has become a core production factor, and enterprises have deposited a large amount of data information in daily operations’ procurement, production, and sales [61]. Executives with digital knowledge experience can give full play to their advantages, implement green innovation, and develop and utilize the data resources embedded in enterprises. Executives with digital knowledge experience can increase the importance and resource allocation of digital innovation activities [62]. By participating in business decisions, executives with digital knowledge experience will transmit the importance of digital concepts to the entire enterprise [63], which will help to bring out the positive effects of digital transformation, thus improving the quality of green innovation.

Third, executives with digital knowledge experience can realize the effective use of green innovation resources and thus improve the efficiency of green innovation. Specifically, executives with digital knowledge can play the expert effect, applying their industry-specific proficiency to address intricate challenges and offering guidance and assistance for digital innovation endeavors [64]. This mitigates the risk of project failure, facilitates the successful conversion of innovation activities into technological accomplishments, and steers the development of green innovation. The widespread application of digital innovation can reduce marginal costs, speed up product updates, help broaden sales channels, obtain external business information in time, and explore new business models [65]. This supports enterprises’ business processes and high-quality green innovation.

**H2: Executives’ digital knowledge experience positively moderates the relationship between digital transformation and green innovation quality**.

### 3.3. The moderating role of intellectual property protection

Intellectual property protection (IPP) enhances the R&D capability of enterprises, which is necessary to maintain their competitive advantage. Intellectual property protection protects the improvement of enterprises’ investment in digital technology and is essential to stimulate digital transformation. This study draws upon a conceptual framework, referred to as "Coleman’s Boat" theory, posited by Coleman in Foundations of Social Theory [66]. The crux of this theoretical perspective contends that institutional and cultural elements (institutional level) at the national echelon can exert an impact on strategic behavioral determinations (behavioral level) by influencing psychological and cognitive biases of individuals (e.g., managers) situated at the microcosmic level.

Drawing on this theoretical framework, even at the same level of digital transformation, the differences in IPP may lead to different perceptions of the cost of digital technology investment, thus different levels of improvement in the quality of green innovation [67]. Specifically, strengthening IPP can change managers’ perceptions of the costs and benefits of digital technology investment because, from the cost perspective, regions with high levels of IPP will reduce knowledge spillover and increase the cost of imitation among enterprises, while reducing the risk of devaluation of enterprises’ digital technology innovation results, and managers expect the digital technology innovation results to be secured [68]. From the revenue perspective, IPP mechanisms can deter patent infringement expeditiously, preclude the financial losses incurred by infringement-related impacts on an enterprise’s digital transformation investments, and enhance the organization’s perceived returns on investment in digital transformation endeavors [69]. Prior research shows that IPP reduces the risk of infringement in the digital transformation process, protects the achievements of digital transformation, and thus increases the value of digital transformation of enterprises [70]. Therefore, IPP is the result of a balanced game between the leverage benefits of digital transformation and the risk costs of increased imitation, and it can serve as a protection mechanism for digital transformation achievements.

As governments implement IPP institutions, the digital transformation trajectory will receive heightened safeguarding, particularly in the context of core green patents. This fosters enterprises’ strategic decision-making concerning digital transformation, ultimately enhancing the quality of green innovation. IPP has the function of exclusivity, and stronger intellectual property protection is conducive to the creation of monopoly profits, effectively safeguarding the excess revenue brought by the digital transformation of enterprises, avoiding to a large extent the infringement of innovators by imitators [71], thus reducing the probability of intellectual property infringement of enterprises, prompting enterprises to enhance the motivation to implement a digital transformation, and laying the foundation for improving the quality of green innovation of enterprises [72]. Therefore, IPP can actualize the augmentation of information proprietary costs, bolster the safeguarding of organizations’ digital transformation accomplishments, and amplify the incentivizing influence of digital transformation on enterprises’ green innovation initiatives. Based on this, the following hypothesis is proposed.

**H3: Intellectual property protection positively moderates the relationship between digital transformation and green innovation quality**.

## 4. Data and methodology

### 4.1. Data and samples

This study selected the data of Chinese A-share listed companies in Shanghai and Shenzhen from 2011 to 2020 as the research sample. The variables related to the degree of digital transformation of companies are obtained from the textual analysis of companies’ annual reports. The biographical information of executives is obtained from the China Stock Market and Accounting Research Database (CSMAR), and other market transactions and financial data are obtained from the WIND database. The CSMAR and WIND databases have been used in prior studies in strategic management and green innovation research [73].

In this study, the data are treated as follows: first, financial firms are excluded; second, special treatment (ST) and particular transfer (PT) since these firms had experienced remarkable financial performance; Third, firms that underwent IPO in the years examined are excluded; fourth, those samples that did not have missing data for at least five consecutive years are retained; fifth, due to the impacts of extreme values, this study winsorizes all continuous variables at the 1% level.

### 4.2. Variable measurement

#### 4.2.1. Dependent variables

Green Innovation Quality (GIQ). In this study, the number of citations of green patents metric is used to take the natural logarithm to measure the quality of green innovation. The number of patent citations reflects patents’ influence and economic value and is a standard indicator of patent quality [74]. Then, the number of patent citations indicates patent quality [75]. The number of patent citations is a vital marker quantity of patent technology influence, and the established literature commonly uses the number of corporate patent citations to portray patent quality [76]. When a green patent is applied for, other green patents that are based on that green patent and then implement green innovation need to be cited in the green patent application. If a green patent is cited more often, it proves that the green patent is more influential in the field. Considering that the longer the green patent is applied for, the higher the probability of its being cited, it is a better solution to use patent citations within five years [77]. Therefore, this study uses the number of green patent citations within five years to portray the quality of green innovation.

#### 4.2.2. Independent variable

Digital Transformation (DT). Referring to the prior research [78], this study reconstructs the DT measure by dividing the frequency of occurrence of the related keywords by the total words of the annual reports of Chinese listed companies used to portray the level of digital transformation. First, the digital transformation feature words are set by combining important policy documents and relevant literature [21]. Second, a large amount of text mining is performed by Python software to extract the frequency of digital transformation-related keywords appearing in the annual reports of enterprises. The number of occurrences of non-company digital transformation words is excluded, including the names of shareholders, executive biographies, and subsidiaries. Finally, the total word frequency of digital transformation in the five dimensions is used. To reduce the influence of the total word count of the annual report text, this study measures the total word frequency of digitization-related words divided by the length of the annual report text.

#### 4.2.3. Moderating variables

Digital Knowledge Experience (DKE). The variable is constructed by identifying executive members with digital technology-related experience in their previous jobs. By manually compiling the executive resumes disclosed in the annual reports of listed companies, this study identifies the digital knowledge experience of executives in terms of their tenure experience. Specifically, executives who had worked in digital technology-related positions or industries before entering their current positions were considered [79]. To identify these positions, their work experience was examined for roles that included terms such as "CIO", "CTO", "information", "computing", "software", "e-commerce", "IT", and other similar digital technology-related terms. For experience in digital-related industries, this study employs the CSMAR industry classification and regards the "software and computer services", "telecommunications services", and "media and entertainment" industries as digital technology-related industries [80]. Finally, an executive with experience in any digital technology-related position or at least three years in a digital technology-related industry was identified. The resulting values were calculated and mean-centered to construct the executive digital knowledge experience variable.

Intellectual Property Protection (IPP). China’s inter-provincial intellectual property protection index is used as a measure, and a more extensive index represents a higher level of regional IP protection. Referring to Brandl et al. (2019) [81] study, the intellectual property protection index from the All-China Intellectual Property Development Report divided by 100 is used to measure the degree of Intellectual property protection.

#### 4.2.4. Control variables

This study has selected the following control variables: total assets (Size), firm age (Age), board size (Board), chairman and general manager (Duality), the proportion of independent directors (Inde), the Shareholding ratio of the largest shareholder (Holder), asset-liability ratio (Lev), cash flow (Cash), Nature of equity (Soe), R&D investment (RD), return on assets (ROA), revenue growth (Growth), and market value (Tobin’s Q). Table 1 presents the descriptive statistics of the variables.

**Table 1:** Variable definitions.

Variables		Description
Dependent Variable	GIQ	The number of citations of green patents metric takes the natural logarithm to measure the quality of green innovation.
Independent Variable	DT	The total word frequency of digitization-related words is divided by the length of the annual report text.
Moderation Variables	DKE	An executive with experience in any digital technology-related position or at least three years in a digital technology-related industry was identified. The resulting values were calculated and mean-centered to construct this variable.
	IPP	The intellectual property protection index from the All-China Intellectual Property Development Report divided by 100 is used to measure the degree of IPP.
Control Variables	Size	The natural logarithm of total assets.
	Age	The natural logarithm of firm establishment time
	Board	Number of the firm board of directors for the year.
	Duality	If the chairman and the CEO are the same person, the value is 1, and the opposite is 0.
	Inde	The proportion of the number of independent directors in the total number of directors
	Holder	The shareholding ratio of the largest shareholder
	Lev	Total corporate debts as a percentage of total assets.
	Cash	The ratio of cash flow from operating activities to total assets at the end of the year
	Soe	When the enterprise is a state-owned enterprise, 1 is taken, otherwise 0 is taken.
	RD	R&D investment to operating income ratio
	ROA	The ratio of net profit after tax to total assets
	Growth	The ratio of the increase in operating revenue of the enterprise at the end of the year to the operating revenue at the beginning of the year
	Tobin’s Q	The market value of a firm’s stock is at the replacement cost of the firm’s total assets

### 4.3. Model design

The research hypotheses are tested using a fixed-effect (FE) model, which establishes the following models:

$$GIQ_{i,t}=\alpha_{0}+\alpha_{l}DT_{i,t}+\alpha_{k}\sum Control_{i,t}+\sum Year+\varepsilon_{i,t}$$ (1)

$$GIQ_{i,t}=\beta_{0}+\beta_{1}DT_{i,t}+\beta_{2}DKE_{i,t}+\beta_{3}DT_{i,t}\times DKE_{i,t}+\beta_{4}IPP_{i,t}+\beta_{5}DT_{i,t}\times IPP_{i,t}+\beta_{k}\sum Control_{i,t}+\sum Year+\varepsilon_{i,t,t}$$ (2)

In Eq (2), *β*_1_ refers to the coefficient of the independent variable (i.e., digital transformation). *Β*_2_ and *β*_4_ represent the coefficients of two moderators (i.e., executive digital knowledge experience, and intellectual property protection). *β*_3_ and *β*_5_ denote the coefficients of two interactions with the dependent variable. *β*_*k*_ represents the coefficients of a series of control variables. ∑ *Year* represent year dummy variables. *ε*_*it*_ is the random error term.

## 5. Empirical results

### 5.1. Descriptive statistics and correlation analysis

Table 2 provides the results of descriptive statistics and correlation tests. the mean value of GIQ is 0.162 and the standard deviation is 0.444, indicating that the level of green innovation quality of enterprises is low, but there is a large difference in the green innovation quality of these enterprises. The mean value and standard deviation of DT are 0.056 and 0.111, respectively, indicating the difference in digital transformation among Chinese A-share firms. The correlation coefficient between DT and green innovation quality is 0.061, which is positively correlated at the 1% level, indicating that digital transformation positively impacts corporate green innovation quality, and H1 has been initially verified. In addition, it can be seen that the standard deviation of the variables tested in this study is relatively large and the correlation coefficient is much less than 0.8, indicating that there is no multicollinearity among the variables and suitable for multiple regression analysis.

**Table 2:** Descriptive statistics and correlation analysis.

Variables	Mean	SD	1	2	3	4	5	6	7	8	9	10	11	12	13	14	15	16	17
1. GIQ	0.162	0.444	1																
2. DT	0.056	0.111	0.061***	1															
3. DKE	0.207	0.234	0.093***	0.243***	1														
4. IPP	0.741	0.125	0.110***	0.081***	0.050***	1													
5. Size	22.288	1.311	0.063***	-0.045***	-0.104***	-0.098***	1												
6. Age	2.896	0.323	-0.149***	-0.031***	-0.086***	-0.157***	0.141***	1											
7. Board	2.247	0.178	0.045***	-0.065***	-0.040***	-0.057***	0.258***	0.034***	1										
8. Duality	0.258	0.437	0.007	0.081***	0.081***	0.090***	-0.166***	-0.089***	-0.189***	1									
9. Inde	0.375	0.054	-0.005	0.047***	0.016**	-0.001	0.002	-0.031***	-0.531***	0.124***	1								
10. Holder	0.359	0.150	0.024***	-0.080***	-0.051***	0.023***	0.171***	-0.132***	0.011*	-0.034***	0.045***	1							
11. Lev	0.441	0.206	-0.029***	-0.108***	-0.137***	-0.089***	0.490***	0.156***	0.143***	-0.117***	-0.006	0.014**	1						
12. Cash	0.045	0.070	0.026***	-0.031***	-0.001	0.017***	0.057***	0.010	0.043***	-0.006	-0.018***	0.109***	-0.168***	1					
13. SOE	0.389	0.487	0.007	-0.122***	-0.076***	-0.180***	0.340***	0.165***	0.277***	-0.291***	-0.069***	0.152***	0.265***	-0.004	1				
14. RD	0.034	0.041	0.105***	0.291***	0.363***	0.105***	-0.242***	-0.136***	-0.138***	0.163***	0.058***	-0.105***	-0.325***	0.015**	-0.259***	1			
15. ROA	0.033	0.061	0.067***	0.029***	0.027***	0.083***	0.036***	-0.068***	0.033***	0.012*	-0.026***	0.161***	-0.330***	0.370***	-0.052***	0.004	1		
16. Growth	0.424	1.163	-0.043***	0.042***	0.008	-0.034***	0.008	0.056***	-0.026***	-0.016**	0.018***	0.002	0.082***	-0.108***	0.027***	-0.001	-0.008	1	
17. Tobin’s Q	2.035	1.352	0.012*	0.149***	0.084***	0.023***	-0.434***	-0.019***	-0.144***	0.083***	0.046***	-0.092***	-0.297***	0.075***	-0.165***	0.230***	0.130***	0.021***	1

Notes: N = 23, 035.

* p < 0.1

** p < 0.05

*** p < 0.01.

### 5.2. The effect of digital transformation on green innovation quality

Table 3 provides regression results for the impact of digital transformation on the quality of green innovation, while including controls for firm and year-fixed effects and controls for relevant variables. In column (1), the DT coefficient is 0.065 and is significantly positive at the 5% level, representing the positive impact of digital transformation on the quality of enterprise green innovation. In column (2), controlling for firm fixed effects, the coefficient of DT on GIQ remains significantly positive, and the finding is consistent with H1.

**Table 3:** The regression results of digital transformation on green innovation quality.

Variables	(1)	(2)
	GIQ	GIQ
DT	0.065**	0.191***
	(2.390)	(6.156)
Size	0.053***	0.060***
	(17.677)	(19.818)
Age	-0.070***	-0.047***
	(-7.005)	(-4.641)
Board	0.023	0.024
	(1.142)	(1.227)
Duality	0.011	0.009
	(1.643)	(1.339)
Inde	0.002	0.010
	(0.032)	(0.154)
Holder	-0.040**	-0.022
	(-2.018)	(-1.143)
Lev	-0.076***	-0.063***
	(-4.222)	(-3.469)
Cash	0.120***	0.102**
	(2.732)	(2.332)
Soe	-0.004	0.015**
	(-0.640)	(2.236)
RD	1.678***	1.110***
	(21.196)	(12.955)
ROA	0.200***	0.152***
	(3.688)	(2.816)
Growth	-0.017***	-0.006**
	(-6.997)	(-2.227)
Tobin’s Q	0.002	0.003
	(0.885)	(1.013)
Constant	-0.890***	-1.123***
	(-11.194)	(-14.075)
Firm FE	No	Yes
Year FE	Yes	Yes
N	23035	23035
Adj.R^2^	0.095	0.120

Notes:

* *p* < 0.1

** *p* < 0.05

*** *p* < 0.01.

Therefore, these empirical results support the hypotheses of this study. These findings suggest that digitally transformed companies tend to be more inclined to pursue sustainability policies and thus improve sustainability performance. Theoretically, the results of this study are consistent with prior sustainability research and agency theory predictions that digital transformation enhances the efficiency of green processes, reduces corporate agency problems, and effectively mitigates agency conflicts between management and shareholders. Enterprises are willing to invest their limited resources in high-quality green innovation activities [82].

### 5.3. Moderating effects

Table 4 provides the regression results for the moderating effects of executive digital knowledge experience and regional IP protection. Columns (1) show the results of the moderating effect of executive digital knowledge experience, and columns (2) show the results of the moderating effect of regional IP protection. Specifically, the moderating term DT×DKE of executive digital knowledge experience is introduced in column (1) to test whether there is a significant linear moderating effect of executive digital knowledge experience. The regression results show that the regression coefficient of the interaction term DT×DKE is 0.223, which is significant at the 5% level, indicating that there is a significant linear moderating effect of executives’ digital knowledge experience, suggesting that the higher the positive relationship between digital transformation and green innovation quality when executives have digital knowledge experience. Therefore, the H2 was supported. Column (2) introduces the moderating term DT×IPP for regional IP protection to test whether regional IP protection has a significant linear moderating effect. The regression results show that the regression coefficient of the interaction term DT×IPP is 0.592, which is significant at the 5% level, indicating that the higher the level of regional IP protection, the stronger the positive relationship between digital transformation and green innovation quality. Therefore, H3 is supported. In addition, column (3) indicates that the two moderating variables are integrated into one model to support the robustness of the study results.

**Table 4:** Moderating effects of executive digital knowledge experience and regional IP protection.

Variables	(1)	(2)	(3)
	GIQ	GIQ	GIQ
DT	0.138***	0.158***	0.103***
	(4.186)	(4.985)	(3.070)
DKE	0.112***		0.109***
	(8.404)		(8.135)
DT×DKE	0.223**		0.243***
	(2.554)		(2.793)
IPP		0.231***	0.227***
		(9.523)	(9.367)
DT×IPP		0.592**	0.601**
		(2.496)	(2.540)
Size	0.061***	0.060***	0.061***
	(20.125)	(19.763)	(20.063)
Age	-0.042***	-0.043***	-0.039***
	(-4.208)	(-4.322)	(-3.915)
Board	0.023	0.031	0.030
	(1.156)	(1.577)	(1.506)
Duality	0.007	0.007	0.005
	(1.083)	(0.993)	(0.746)
Inde	0.013	0.030	0.033
	(0.207)	(0.488)	(0.537)
Holder	-0.020	-0.028	-0.026
	(-1.045)	(-1.424)	(-1.325)
Lev	-0.067***	-0.060***	-0.064***
	(-3.680)	(-3.315)	(-3.534)
Cash	0.112**	0.093**	0.103**
	(2.564)	(2.110)	(2.342)
Soe	0.011	0.024***	0.020***
	(1.638)	(3.491)	(2.882)
RD	0.963***	1.051***	0.908***
	(11.068)	(12.256)	(10.433)
ROA	0.134**	0.125**	0.108**
	(2.477)	(2.323)	(1.996)
Growth	-0.006**	-0.005*	-0.005**
	(-2.350)	(-1.853)	(-1.976)
Tobin’s Q	0.003	0.003	0.004
	(1.192)	(1.307)	(1.476)
Constant	-1.150***	-1.154***	-1.176***
	(-13.518)	(-13.565)	(-13.847)
Firm FE	Yes	Yes	Yes
Year FE	Yes	Yes	Yes
N	23035	23035	23035
Adj.R^2^	0.124	0.124	0.127

Notes:

* *p* < 0.1

** *p* < 0.05

*** *p* < 0.01.

### 5.4. Endogeneity test

2SLS regression method. This study may be concerned that the findings are influenced by endogeneity issues caused by the reverse causality between digital transformation and the quality of green innovation. For example, enterprises that aspire to improve the quality of green innovation are more likely to implement digital transformation. Therefore, two-stage least squares (2SLS) estimation is conducted to mitigate the endogeneity problem further. The following IV was used in this study: the MeanIndDT. MeanIndDT was defined as an indicator of the degree of digital transformation of other enterprises in the same industry in the previous period, as an instrumental variable. This study argues that firms are more likely to implement digital transformation if this is the industry norm [83]. In addition, MeanIndDT is unlikely to affect individual firms’ green innovation quality and therefore satisfies the exclusion criteria for instrumental variables.

The results of the endogeneity tests are reported in Table 5. The coefficient of the predictive value of the variable DT remains significant in the second-stage regression, indicating support for a positive correlation between digital transformation and the quality of green innovation after accounting for endogeneity issues.

**Table 5:** Endogeneity test result.

Variables	(1)	(2)
	First stage	Second stage
	DT	GIQ
DT		0.253***
		(5.628)
MeanIndDT	0.716***	
	(143.781)	
Size	0.001	0.056***
	(0.393)	(18.524)
Age	0.001	-0.055***
	(0.424)	(-4.995)
Board	0.007**	0.027
	(2.155)	(1.310)
Duality	0.002	0.005
	(1.564)	(0.693)
Inde	0.036***	-0.012
	(3.473)	(-0.189)
Holder	-0.002	-0.020
	(-0.506)	(-0.974)
lev	-0.004	-0.032*
	(-1.286)	(-1.657)
Cash	0.003	0.110**
	(0.469)	(2.298)
Soe	-0.004***	0.012*
	(-3.384)	(1.736)
RD	0.102***	1.211***
	(7.272)	(13.208)
ROA	0.036***	0.308***
	(3.867)	(5.175)
Growth	0.020***	-0.049***
	(10.048)	(-3.929)
Tobin’s Q	0.001***	0.002
	(3.641)	(0.749)
Constant	-0.032**	-1.262***
	(-2.349)	(-14.012)
Firm FE	Yes	Yes
Year FE	Yes	Yes
N	19607	19607
Adj.R^2^	0.689	0.125

Notes:

* *p* < 0.1

** *p* < 0.05

*** *p* < 0.01.

### 5.5. Robustness test

First, they are replacing measures of firms’ green innovation quality. This study refers to the previous literature and uses the logarithm of the sum of the number of patents filed for green inventions plus one to remeasure the quality of green innovation [84], because of the more stringent approval of green invention patent applications and higher technical level characteristics. The regression results are shown in Table 6, from which it can be seen that digital transformation is positively related to the quality of green innovation of firms, a result that is not significantly different from the regression results described in the previous sections, indicating that the results of this study are robust.

**Table 6:** Robustness test of the replacement of the measurement method of green innovation quality.

Variables	(1)	(2)
	GPatent	GPatent
DT	0.322***	0.592***
	(7.133)	(11.573)
Size	0.157***	0.173***
	(31.355)	(34.408)
Age	-0.112***	-0.058***
	(-6.703)	(-3.490)
Board	0.104***	0.078**
	(3.108)	(2.361)
Duality	0.011	0.012
	(0.992)	(1.066)
Inde	0.069	0.011
	(0.656)	(0.103)
Holder	-0.129***	-0.093***
	(-3.922)	(-2.877)
Lev	0.167***	0.188***
	(5.578)	(6.228)
Cash	0.156**	0.079
	(2.138)	(1.081)
Soe	0.027**	0.049***
	(2.410)	(4.426)
RD	4.105***	3.137***
	(31.160)	(22.156)
ROA	0.632***	0.553***
	(6.998)	(6.204)
Growth	-0.017***	0.007*
	(-4.254)	(1.661)
Tobin’s Q	0.014***	0.016***
	(3.294)	(3.795)
Constant	-3.372***	-3.814***
	(-23.923)	(-27.108)
Firm FE	No	Yes
Year FE	Yes	Yes
N	22944	22944
Adj.R^2^	0.119	0.156

Notes:

* *p* < 0.1

** *p* < 0.05

*** *p* < 0.01.

Second, this study uses the method of replacing regression models for robustness testing, we rerun the model in Table 7 using Tobit’s and Poisson’s methods. This study’s results are similar to earlier analyses (available upon request) and support the positive impact of digital transformation on the quality of green innovation.

**Table 7:** Robustness test of the replacement regression model.

Variables	(1)	(2)
	Tobit Model	Poisson Model
	GIQ	GIQ
DT	0.895***	0.860***
	(4.738)	(5.698)
Size	0.449***	0.345***
	(20.634)	(20.894)
Age	-0.231***	-0.192***
	(-3.580)	(-3.493)
Board	0.132	0.093
	(0.979)	(0.825)
Duality	0.015	0.034
	(0.337)	(0.860)
Inde	-0.470	-0.426
	(-1.137)	(-1.158)
Holder	-0.321**	-0.191
	(-2.383)	(-1.630)
Lev	-0.308**	-0.251**
	(-2.328)	(-2.184)
Cash	0.545*	0.446
	(1.675)	(1.543)
Soe	0.023	0.027
	(0.502)	(0.656)
RD	8.086***	6.209***
	(14.671)	(15.147)
ROA	2.128***	1.979***
	(4.941)	(4.998)
Growth	-0.097***	-0.079***
	(-3.864)	(-3.444)
Tobin’s Q	-0.014	-0.010
	(-0.747)	(-0.669)
Constant	-11.345***	-9.277***
	(-18.629)	(-18.417)
Year FE	Yes	Yes
N	23035	23035
Pseudo.R^2^	0.155	0.191

Notes:

* *p* < 0.1

** *p* < 0.05

*** *p* < 0.01.

Third, consideration of the time lag effect. To circumvent the endogeneity effect in the relationship between digital transformation and the quality of corporate green innovation, this study uses lagged digital transformation (LagDT) data for empirical analysis, with results shown in Table 8. The column (2) shows a significant positive correlation between LagEID and the quality of corporate green innovation, suggesting that digital transformation enhances the quality of corporate green innovation. These results demonstrate robustness.

**Table 8:** Robustness test of the one-period lagged DT (LagDT).

Variables	(1)	(2)
	GIQ	GIQ
LagDT	0.048*	0.181***
	(1.694)	(5.535)
Size	0.049***	0.056***
	(14.941)	(16.862)
Age	-0.078***	-0.054***
	(-6.969)	(-4.745)
Board	0.024	0.027
	(1.126)	(1.280)
Duality	0.005	0.003
	(0.645)	(0.442)
Inde	-0.007	0.007
	(-0.101)	(0.105)
Holder	-0.039*	-0.019
	(-1.823)	(-0.871)
Lev	-0.049**	-0.036*
	(-2.495)	(-1.789)
Cash	0.154***	0.132***
	(3.185)	(2.738)
Soe	-0.004	0.014**
	(-0.552)	(2.004)
RD	1.811***	1.231***
	(21.195)	(13.209)
ROA	0.278***	0.230***
	(4.783)	(3.972)
Growth	-0.017***	-0.005**
	(-6.451)	(-1.979)
Tobin’s Q	0.002	0.002
	(0.581)	(0.892)
Constant	-0.799***	-1.048***
	(-8.624)	(-11.232)
Firm FE	No	Yes
Year FE	Yes	Yes
N	19125	19125
Adj.R^2^	0.101	0.126

Notes:

* *p* < 0.1

** *p* < 0.05

*** *p* < 0.01.

### 5.6. Further research

This study will further test the intrinsic mechanism of digital transformation to enhance the quality of corporate innovation. According to the analytical logic of the research hypotheses, it is believed that digital transformation promotes green innovation quality through a consumer needs perspective, enhancing information transparency, and improving resource allocation efficiency. Therefore, this study will investigate the above three mechanisms. According to the availability of corporate data, the selection and data processing of the above three indicators are as follows.

First, green business income (Green_income). Referring to prior research [85], because the greater consumer demand for green products will increase the greener consumption income of enterprises, this study selects green operating income as a proxy variable for consumer demand. Specifically, the percentage of green revenue in this paper is categorized from the perspective of income, using the business composition of listed companies to match with the green industry catalog, and then get the proxy indicator of green operating revenue.

Second, information transparency (Infor_trans). Referring to prior research [86], the degree to which external information users can effectively access specific information (such as annual reports, various disclosure announcements, analysts’ reports, and information voluntarily disclosed by companies) of a publicly traded listed company is adopted, and information transparency is calculated from indicators such as surplus quality, disclosure appraisal index, analysts’ surplus forecasts, and auditor’s point of view to measure the company.

Third, resource allocation efficiency (TFP). Referring to prior research [87], this study adopts the total factor productivity (TFP) index to measure resource allocation efficiency. This study adopts the LP method to estimate total factor productivity, and the larger the indicator is, the higher the resource allocation efficiency.

The results of the intrinsic mechanism test for the above three levels are shown in Table 9. After controlling for firm and year fixed effects, the empirical results show that digital transformation has a significantly positive impact on green operating revenue (coef. = 0.499, p < 0.01), and a significantly positive impact on firm information transparency and total factor productivity (coef. = 0.417, p<0.01; coef. = 0.656, p<0.01). The empirical test results confirm that digital transformation can improve the quality of corporate green innovation by increasing corporate green operating income through enhancing green consumption and other ways, improving corporate information transparency, and increasing corporate resource allocation efficiency.

**Table 9:** Mechanism testing of digital transformation on green innovation quality.

Variables	(1)	(2)	(3)
	Consumer needs	Information transparency	Resource allocation efficiency
	Green_income	Infor_trans	TFP
DT	0.499***	0.417***	0.656***
	(12.022)	(4.633)	(16.131)
Size	0.922***	-0.041***	0.643***
	(226.728)	(-4.678)	(158.794)
Age	-0.026*	-0.533***	-0.035***
	(-1.960)	(-18.217)	(-2.646)
Board	-0.007	0.070	-0.093***
	(-0.271)	(1.208)	(-3.490)
Duality	-0.028***	0.098***	-0.016*
	(-3.119)	(5.037)	(-1.867)
Inde	-0.158*	-0.106	-0.229***
	(-1.903)	(-0.590)	(-2.774)
Holder	0.242***	0.049	0.155***
	(9.240)	(0.866)	(5.915)
Lev	0.808***	-0.517***	0.738***
	(33.047)	(-9.740)	(30.323)
Cash	1.388***	-0.061	0.643***
	(23.585)	(-0.475)	(10.962)
Soe	0.090***	-0.272***	0.043***
	(9.983)	(-13.894)	(4.771)
RD	-3.639***	3.008***	-3.266***
	(-31.703)	(12.073)	(-29.014)
ROA	2.055***	3.324***	2.280***
	(28.413)	(21.180)	(31.942)
Growth	-0.042***	-0.014*	-0.018***
	(-12.427)	(-1.935)	(-5.347)
Tobin’s Q	-0.000	-0.046***	0.009***
	(-0.075)	(-6.375)	(2.823)
Constant	0.688***	4.804***	-5.207***
	(6.034)	(19.420)	(-45.917)
Firm FE	Yes	Yes	Yes
Year FE	Yes	Yes	Yes
N	21738	21738	21738
Adj. R^2^	0.851	0.269	0.765

Notes:

* *p* < 0.1

** *p* < 0.05

*** *p* < 0.01.

## 6. Conclusion

Digital transformation is important for enterprises to achieve green high-quality innovation development. This study constructs digital transformation indicators at the enterprise level by crawling the keywords related to digital transformation in the annual reports of Chinese-listed companies. This study empirically examines the impact of digital transformation on the quality of green innovation and the role of internal and external knowledge as boundary conditions, using a sample of Chinese-listed companies from 2011 to 2020. It is found that digital transformation improves the quality of green innovation, indicating that sustainability-oriented digital transformation has a driving role in driving the quality of green innovation. In addition, this study examines how executive discretion is influenced by digital knowledge experience and regional intellectual property protection. Specifically, the positive relationship between digital transformation and green innovation quality is stronger when executives have a higher digital knowledge experience and regional intellectual property protection with higher discretionary power. The study of the impact of digital transformation on the quality of corporate green innovation can provide suggestions for high-quality green development in countries with emerging economies.

## 7. Research contribution

### 7.1. Theoretical implications

The study contributes to the literature in these ways. First, this study provides a novel digital transformation framework to explain its impact on the quality of green innovation. Firms rely on digital technologies to address environmental issues [88]. Prior studies have focused on the impact of digital technology on corporate environmental performance, by analyzing how it optimizes resource management processes [89]. Others have called for more research on how digitalization can achieve environmental sustainability [90]. This result provides novel ways of thinking about the impact of digital transformation and explains why managers should engage in green innovation. Moreover, the findings of this study indicate that effective management and response to consumers’ green demands and assertions, facilitated by digital transformation, cater to consumers’ green preferences in line with the concept of green sustainable development. This encourages firms to focus on high-quality green innovation, aiming to achieve an interactive inclination between consumers’ green preferences and corporate high-quality green innovation. Consequently, this study’s results propel the argument on how consumers can exert pressure on digitally transforming firms to implement green initiatives for maintaining competitiveness.

The second salient contribution is scrutinizing internal and external knowledge dynamics and their moderate roles. Regarding the internal executive knowledge role, this study finds an essential moderating role for executives with digital knowledge experience in the relationship between digital transformation and green innovation quality. The research contributes to the study of the role of executives and the competencies required for digital transformation [91]. Specifically, case studies and conceptual research on digital transformation suggest that executives understand and support a firm’s digital transformation efforts, but it is not clear what specific characteristics executives need. The study adds empirical evidence to this literature by outlining that firms’ digital transformation can benefit from digital knowledge among executives. This study informs the debate on whether leadership in the digital age requires general managerial competencies or digital competencies [91]. This study supports the view in recent conceptual works on digital transformation that a digital technology-centric approach to management is becoming important.

Further, on the effectiveness of regions with high-level IPP to enhance the quality of digital transformation for green innovation. Scholars imply that the lack of an IP protection-driven regime is one of the most significant barriers to digital transformation [92]. This study confirms this logic by providing empirical evidence of the ability of firms under IPP to harness the potential of digitalization for green innovation. Research has demonstrated that regions with more developed IPPs are likely to have higher labor productivity and lower information search costs [90], increasing the efficiency of green resource management. Thus, combining IPP in regional settings with broader green digitization would likely encourage managers to engage in green innovation. This finding highlights the importance of an enterprise’s involvement in reducing resistance and promoting digital effectiveness. Given that high-quality green innovation requires significant resource investments, these findings are consistent with the logic of digital innovation research, which suggests that modest resource support for digital transformation is needed to improve the quality of green innovation [93].

### 7.2. Managerial implications

The practical contributions of these findings are discussed below. First, enterprises promote digital transformation and the quality of green innovation. The rapid development of digital technology has brought fundamental changes to enterprises in the traditional model of innovation being overturned. Enterprises should optimize the ability of digital transformation, increase the investment in the process of enterprise operation, and reduce the cost of enterprise operation through digital technology, to enhance the positive impact effect of digital transformation on the quality of green innovation.

Second, managers have an important role in digital transformation in companies due to the relevance of digital transformation and the debate on the digital knowledge characteristics of executives. The study confirms that executives’ digital knowledge experience has an important role in advancing digital transformation, that they are better able to perform their emerging digital transformation tasks through their experience and resources, and that managers should promote corporate digital transformation by devoting more resources to green innovation activities, such as identifying the potential of digital transformation and supporting its implementation. Therefore, companies should consider the relevance of digital knowledge in the design of executive composition processes and leadership development programs (e.g., training, workshops, etc.).

Third, digital transformation enhancement lies in the external IPP role. With the booming digital economy, companies should pay more attention to protecting their intellectual property rights to maintain a greater advantage in the competition of information technology and digitalization, and become an essential driving force for the green innovation effect of digital transformation. Therefore, to achieve enterprise success, enterprises’ green innovation quality should be improved by improving the institution of regional IPP based on digital transformation. In conclusion, enterprises focus on implementing enterprise digital transformation to realize the double-wheel drive phenomenon of "digital transformation" and "enterprise green innovation development".

### 8. Limitations and future research

The study highlights several limitations that require further research. For the measurement of green innovation quality, green patents are used in this study, but in highly polluting enterprises, updating equipment and processes may reduce pollution, which belongs to green innovation, but cannot be measured by green patents. Therefore, green patents do not represent green innovation. However, since specific data on the plant equipment of enterprises are not available, green patents are used in the existing literature to measure green innovation [94, 95]. It is hoped that more accurate metrics for measuring green innovation will be found in the future.

Second, this study considers the moderating role of environmental factors (i.e., regional intellectual property protection regimes) and executive characteristics (i.e., executives’ digital knowledge experience). In terms of future research directions regarding moderating mechanisms, other factors include government policies, institutional vulnerability, pollution intensity, and board structure [96]. These factors may be moderating variables in the relationship between digital transformation and green innovation quality and can be further investigated.

Third, the context of this study is China, a country undergoing economic reform and environmental transformation, which may limit the generalizability of our findings to other contexts, such as the United States. This limitation is because these two countries have different economic, political, and cultural levels. Therefore, this study calls for a comparative analysis of digital transformation to gain insight into its impact on the quality of green innovation relative to these context-specific characteristics.

## Supporting information

S1 Data (XLSX)
